# A pilot study on acute inflammation and cancer: a new balance between IFN-γ and TGF-β in melanoma

**DOI:** 10.1186/1756-9966-28-23

**Published:** 2009-02-19

**Authors:** Yue-mei Ma, Tao Sun, Yi-xin Liu, Nan Zhao, Qiang Gu, Dan-fang Zhang, Shuo Qie, Chun-sheng Ni, Yi Liu, Bao-cun Sun

**Affiliations:** 1 Department of Pathology, Tianjin Cancer Hospital, Tianjin Medical University, Tianjin, PR China; 2 Department of Pathology, Tianjin Medical University, Tianjin, PR China; 3 Department of Operative Surgery, Tianjin Medical University, Tianjin, PR China

## Abstract

Recent data have redefined the concept of inflammation as a critical component of tumor progression. However, there has been little development on cases where inflammation on or near a wound and a tumor exist simultaneously. Therefore, this pilot study aims to observe the impact of a wound on a tumor, to build a new mouse tumor model with a manufactured surgical wound representing acute inflammation, and to evaluate the relationship between acute inflammation or wound healing and the process of tumor growth. We focus on the two phases that are present when acute inflammation influences tumor. In the early phase, inhibitory effects are present. The process that produces these effects is the functional reaction of IFN-γ secretions from a wound inflammation. In the latter phase, the inhibited tumor is made resistant to IFN-γ through the release of TGF-β to balance the inflammatory factor effect on the tumor cells. A pair of cytokines IFN-γ/TGF-β established a new balance to protect the tumor from the interference effect of the inflammation. The tumor was made resistant to IFN-γ through the release of TGF-β to balance the inflammatory effect on the tumor cells. This balance mechanism that occurred in the tumor cells increased proliferation and invasion. *In vitro *and *in vivo *experiments have confirmed a new view of clinical surgery that will provide more detailed information on the evaluation of tumors after surgery. This study also provides a better understanding of the relationship between tumor and inflammation, as well as tumor cell attacks on inflammatory factors.

## Introduction

As early as 1863, Virchow first postulated that cancer originates at the sites of chronic inflammation. This is partly based on his hypothesis that some classes of irritants causing inflammation also enhance cell proliferation [1]. In the past decades, scientists have made considerable progress in research on the relationship between cancer and inflammation [1,2]. Inflammation is a part of the host's response to either internal or external environmental stimuli. This response serves to counteract the trauma incurred by these stimuli against the host. This can be pyrogenic, as indicated by fever. Acute inflammation or fever manifested for a short period has a therapeutic consequence [3]. Under normal circumstances, the wound healing process is considered an acute inflammation, like surgical wound healing. This process involves the classic model of inflammatory response, including the formation of granulation tissue, leukocyte infiltration, angiogenesis factor, and cytokines network [4]. During acute inflammation, the emergence of cell proliferation, angiogenesis, and reconstruction of the organization are very similar to tumor growth and progression. However, since there are two different consequences, the former is limited and controlled, while the latter is unlimited and irregular [2,5].

Tumor patients are characterized with an abnormal immune function, with majority of the patients having low immunity. Some have enforced incomplete tumor resection where the surgery wound also heals normally. This is a common clinical phenomenon. In tumor cells that can secrete a strong cytokines pattern, the residual tumor cells particularly release a large amount of cytokines after incomplete resection, which stimulate the surrounding tissue under repair, thus speeding up the wound healing process. This involves the vascular endothelial growth factor (VEGF), epidermal growth factor (EGF), basic fibroblast growth factor (bFGF), and other cytokines [3,6]. This makes the question on whether the influence of surgery wounds on tumor cells depresses or promotes proliferation in tumor cells, interesting.

To evaluate the relationship between acute inflammation or wound healing and tumor growth, this study utilizes a mouse tumor model with a manufactured surgical wound. The model is capable of building a representation of acute inflammation. Present in this model are the inhibitory effects on tumor growth of acute inflammation in the early stage, which is the functional reaction of IFN-γ due to wound inflammation. In the latter stage, the role of the tumor is to resist IFN-γ by releasing TGF-β to balance the inflammatory factor effect on the tumor cells. Similar to real situations, a pair of cytokines IFN-γ/TGF-β established a new balance to protect the tumor from the interference factor of inflammation. Likewise, a new immune escape mechanism in the tumor cells occurred because of increased access to cell proliferation. Our in vitro and in vivo experiments confirmed a new view of clinical surgery that will provide more detailed information to evaluate tumors after surgery. The study also offers a better understanding of the relationship between tumor and inflammation, as well as tumor cells and attacks on immunity.

## Materials and methods

### Cells and Animals

Cells: Mouse melanoma cell-line B16F10 was supplied by the Department of Cell Biology, Huanhu Hospital, Tianjin, People's Republic of China. The cell was cultured in RPMI1640 medium (Hyclone) containing 10% fetal bovine serum (FBS: Gibco), 50 units/ml penicillin, and 50 μg/ml streptomycin (Gibco). In all the experiments, the cell was maintained in 100 mm culture dishes (Costar) at 37°C in humidified 5% CO_2_/95% air atmosphere.

Animals: female six-week old, 18~22 g C57/BL mice were purchased from the Animal Center Academy of Military Medical Science (License: SCXK [Jin] 2004-0001; Beijing, China). They were brought to the Animal Centre of Tianjin Medical University one week before the experiment and were bred under the specific pathogen-free (SPF) conditions.

### Proliferation Assay

(Sulforhodamine-B, Sigma) (SRB) assay: Exponentially growing B16F10 melanoma cell lines were plated at 5 × 10^4 ^cells/ml in flat-bottomed 24-well plates (Costar) in 1 mL of complete RPMI 1640 medium containing 1% fetal bovine serum. The tumor cells were then incubated for 8 h, 16 h, 24 h, 32 h, 40 h, and 48 h in various concentrations of cytokines at a total volume of 1 ml. The final concentration of TGF-β (Peptech) was 5 ng/ml, while that of IFN-γ (Peptech) was 10 ng/ml. The solution without cytokines was assigned as the control group. After incubation for a specific number of hours at 37°C in 5% CO_2_, fixing, and staining by SRB, the optical densities and percentage viability were then determined by absorption at 540 nm (A540).

### Invasion and Wound Healing Assay

Migration assay was performed using Transwell cell culture inserts with 8 μm porosity polyethylene teraphthalate filters (Invitrogen). Briefly, confluent tumor cells were trypsinized and plated onto the upper Matrigel-coated insert and were allowed to attach to the membrane for 1 h. Cytokines were then added into the free-FBS media in the upper inserts, free-cytokines, and free-FBS media as controls. The lower inserts used 20% FBS media as chemoattractant both in the cytokines groups and the controls. Cells were allowed to migrate for 24 h. The upper surface of the membrane was then wiped to remove nonmigratory cells. The cells that invaded through the Matrigel and adhered to the bottom of the membrane were stained with crystal violet solution. The cell-associated dye was eluted with 10% acetic acid, and its absorbance at 595 nm was determined. Each experiment was done in triplicate, and the mean values, mean ± SE were presented. Wound healing assays were done with six-well chambers. Cell motility was assessed by measuring the movement of the cells into a scarped, acellular area created by a 200 μL pipette tube (time 0). The speed of the wound closure was monitored after 12 h, and the ratio of the distance of the wound in relation to 0 h was measured. Each experiment was also done in triplicate, and the mean values, mean ± SE were presented.

### Mouse Tumor with Wound Model

About 10^7^/mL B16F10 melanoma cell suspension was injected into the left groin area of the mice in 0.2 ml for each mouse. Thirth-one mice were randomly divided into the wound group (18 mice) and the control group (13 mice). Ten days after the tumor cells were engrafted, the tumor masses were detected. When the tumor grew to 1 cm^3^, a 2~3 cm^2 ^wound was built on the opposite side of a bodies in the wound group. The mice in the control group were treated without wounds. The mice were sacrificed by cervical decapitation on the seventh and 11th days following continuous wound treatment.

### Mouse Tumor Model with Cytokines injection

A volume of 0.2 ml of about 10^7^/mL B16F10 melanoma cell suspension was injected into the left groin area of the mice. Sixteen mice were randomly divided into the wound group (8 mice) and the control group (8 mice). When the tumor of the mice in the wound group grew to 5 cm^3^, they were injected with IFN-γ (500 pg/g) through the tail-vein every 3 days. The mice in the control group were only treated with 0.9% NaCl solution. Then the mice were sacrificed by cervical decapitation on the 7th and 11th days following continuous wound treatment.

### Gelatin Zymography

Gelatin zymography was used to examine the levels of matrix metalloproteinases-2 (MMP-2) and MMP-9 activity after the cells were treated with cytokines. To change all media into free-FBS conditioned media and replace the treated cells with cytokines after 24 h, the free-FBS conditioned media were used as a control. All media were collected and subjected to sodium dodecyl sulfate polyacrylamide gel electrophoresis (SDS-PAGE) using 0.01% w/v gelatin containing 10% polyacrylamide gel. After electrophoresis, the gels were equilibrated in 50 mM Tris-HCl (pH 7.5) with 2.5% Triton X-100 for 30 min at room temperature. They were then incubated in 50 mM Tris-HCl (pH 7.5), 10 mM CaCl_2_, 150 mM NaCl, 1 mM ZnCl_2 _and 0.02% NaN_3 _for 20 h at 37°C. The gels were stained with Coomassie R250 and destained until the wash became clear and the cleared zones associated with MMP activity were apparent. The zymogram was digitized and the amount of clearing associated with MMP-2 and MMP-9 activity was determined using the Gene Genius Super system. The values were calculated using densitometry. Samples of the animals' tumor were lysed by 2% SDS in liquid nitrogen, and the lysates were collected and centrifuged to obtain soluble cell extract.

### Immunohistochemical Staining Methods

Four micrometer-thick sections were mounted on poly-L-lysine-coated slides. Slides were deparaffinized in xylene. Endogenous peroxidase (POD) activity was blocked with 3% hydrogen peroxide in 50% methanol for 10 min at room temperature. Sections were rehydrated in alcohol, washed with phosphate-buffered saline (PBS) and then pretreated with citrate buffer (0.01 M citric acid, pH 6.0) for 20 min at 95°C in a microwave oven. After nonspecific binding sites were blocked by exposing them to 10% normal goat serum in PBS for 20 min at 37°C, sections were incubated overnight at 4°C with a series of antibodies (Santa Cruz Biotechnology, dilution 1:100). Following this incubation, the sections were rinsed with PBS and incubated with biotinylated goat anti-mouse IgG for 20 min at 37°C. The slides were then incubated with 3, 3'-diaminobenzidine chromogen for 5–10 min at room temperature and washed with distilled water. Finally, sections were slightly counterstained with hematoxylin for 1 min followed by dehydration and coverslip mounting. PBS was utilized in place of the primary antibodies for the negative control. The staining systems used in this study were PicTure PV6000 (Zhongshan Chemical Co., Beijing, China) and Elivision Plus (Zhongshan Chemical Co. Beijing).

### Enzyme-linked immunosorbent assay (ELISA) Analysis of the Levels of IFN-γ and TGF-β

The IFN-γ and TGF-β detection ELISA kit was used to detect the concentration of IFN-γ and TGF-β in the mouse model serum and the tumor lysate, in accordance with operating manuals. The reaction between POD and ABTS was photometrically determined using a microplate reader at 405 nm.

### Statistical Analysis

All data in the study were evaluated using SPSS11.5 (SPSS Inc., USA). Differences were considered significant at values of p < 0.05. Significant results were marked with "*".

## Results

### Inflammation effect on the melanoma showed two phases: Inhibition and inhibition missing

**Figure 1:**
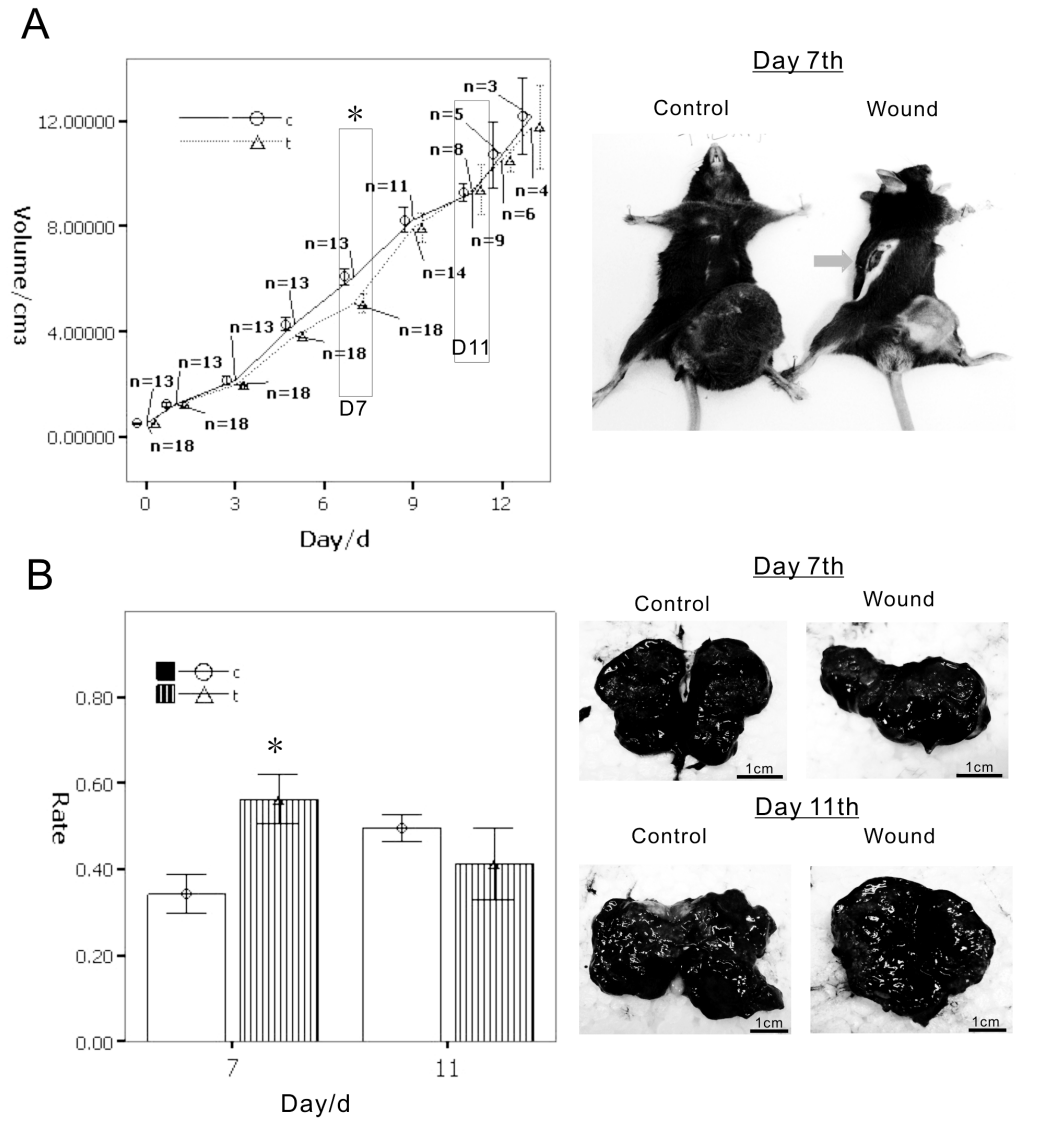
**A wound model was built in C57BL/B16 tumor-bearing mouse to determine the influence on melanoma by inflammation**. When the tumor grew to 0.5 cm^3^, we created a wound beyond the tumor in the opposite site of the mouse's body. A.) The results show gradual reduction of the tumor volume when the wound was building; the tumor volume reached the minimum at day 7 (shown in black box, *p *< 0.01). After day 7, the tumor inhibitory effect of the wound (inflammation) weakened gradually. On about day 11 of the inflammatory group compared with the control group, tumor volume almost as same as the control group at day 13 (shown in black box, *p *> 0.05). B.) The cross-section of the tumor showed that the tumor necrosis with hemorrhage occurred in different proportions of times and groups. On day 7, the group wound tumors were smaller than the control group, and the area with necrotic tissue is greater than the control group (*p *< 0.01). After 11 days, the tumor volume in the wound group was increased, but in the cross-section area of necrotic tissue rather than in the control group (*p *> 0.05). The necrotic percentage after day 11 showed the tumor through a mechanism to adapt the wounds caused by inflammation induced necrosis, promoted the emergence of proliferation.

To determine if inflammation has an inhibitory effect on the melanoma cells, a wound mouse model was built. When the tumor grew to a specific size, we created a wound in the opposite side of the mouse's body. The wound model was used to manufacture a full-body model of acute inflammation in order to investigate the macro effect between inflammation and tumors. The results show a gradual reduction of tumor volume when the wound was building; the tumor volume reached the minimum at day 7. After day 7, the inhibitory effect of the wound (inflammation) on the tumor down-regulated gradually. The tumor volume of the inflammatory group at day 11 was almost the same as the control group at day 13. This is even higher than the average tumor volume. The tumor growth curve showed two phases: the early phase (before day 7, the inhibition phase) and the latter phase (after day 7 and marked in day 11, the inhibition missing phase). The latter phase presented an increasing proliferation of tumors. (Figure 1A)

The cross-section of the tumor showed that the tumor necrosis with hemorrhage occurred at different times and groups. On day 7, the tumors in the wound group were smaller than those in the control group, while the areas with necrotic tissues were greater than those in the control group. After 11 days, the tumor volume in the wound group was increasing, but the necrotic areas in the cross-section decreased in a faster rate than those in the control group. The necrotic percentage after day 11 showed that the tumor, through a mechanism to adapt to the wounds caused by inflammation, induced necrosis which promoted proliferation (Figure 1B). These results indicate that in the early phase, the inflammation occurred, and the inflammatory factors secreted into the blood indirectly influenced the tumor and induced necrosis so that the tumor regressed. In the latter phase, although inflammation was still present, biological changes gave the tumor the ability to resist inflammation, and even enhanced the ability of the tumor cells to increase.

### New balance in inflammation and melanoma: the lever roles of IFN-γ/TGF-β

To further observe and determine the other inflammatory factors in the interaction between tumors and inflammation, we collected the serum samples used to screen the cytokines. The results showed that the level of IFN-γ in the serum for the wound group continued at high levels of expression. High concentrations of IFN-γ were also detected in the tumor tissue. IFN-γ is an inflammation factor mainly because of the secretions of the Th1 cells. It inhibits tumor activity via the normal physiological process for cell death [7,8]. We also conducted an analysis on the other inflammatory factors in our experiment, such as interleukin-1(IL-1), IL-4, IL-10, tumor necrosis factor-α(TNF-α), and vascular endothelial growth factor-a (VEGF-a) which were not observed as influential to the tumor growth curve (data not shown). However, the results show that IFN-γ's inflammatory factor has an impact on tumor tissue, inhibits tumor growth, and induces tumor cell apoptosis or necrosis.

**Figure 2:**
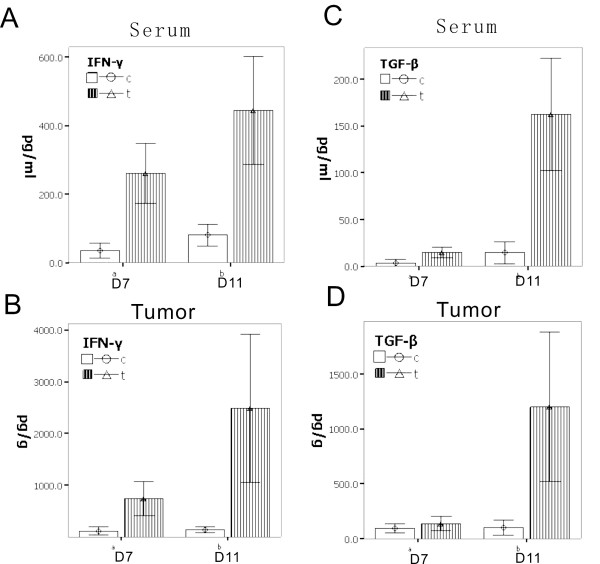
**To further observe and determine the inflammatory factors in the interaction between tumor and inflammation, results showed that: A.) the level of IFN-γ in the serum in the wound group continued a high level of expression (day 7 *p *< 0.01, day 11 *p *< 0.01); B.) in tumor tissue also detected high concentrations compared with the control group (day 7 *p *< 0.01, day 11 *p *< 0.01)**. Interestingly, at the 11th day, the tumor with the TGF-β increased, the result is that: C.) high levels of TGF-β can also be detected in the serum (day 7 *p *> 0.05, day 11 *p *< 0.01); D.) the same change in tumor (day 7 *p *> 0.05, day 11 *p *< 0.01). The TGF-β level before day 7 is not clear in terms of the present low of expression and secretion of tumor cells. That showed that at this time, the tumor does not have to go through the regulation of TGF-β to go against the ability of IFN-γ. When the IFN-γ-induces inhibition of tumor necrosis and persistence over a period, the role of TGF-β has been demonstrated, giving the tumor cells the ability to fight against the IFN-γ, so that the tumor cells could grow.

Interestingly, after day 7, TGF-β increased in the tumors. The TGF-β level before day 7 day was detected in the category of low expression and secretion of tumor cells (Figure 2). Figure 2 shows that the tumor has to enhance the regulation of TGF-β to fight against IFN-γ. The role of TGF-β has been demonstrated with the IFN-γ-induced inhibition of tumor necrosis and persistence over a period, giving tumor cells the ability to fight IFN-γ and thus resulting in tumor cell growth.

### Investigation of the antagonism between IFN-γ and TGF-β in vitro

**Figure 3:**
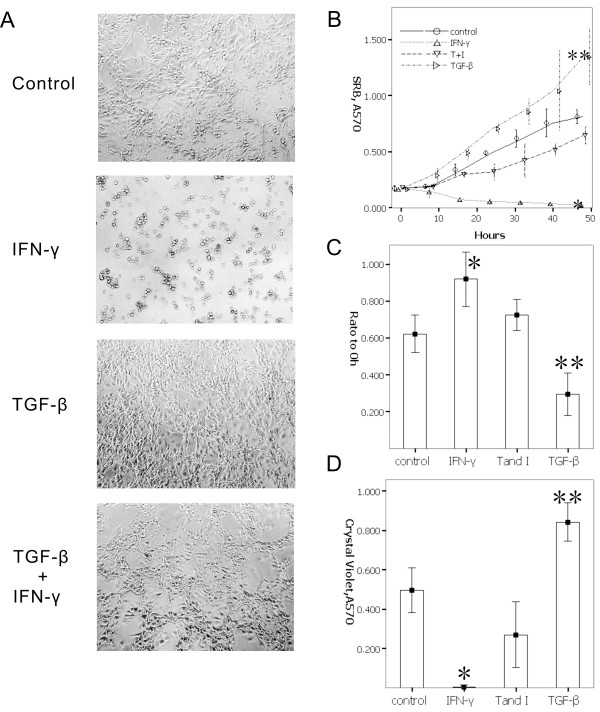
**To investigate the cells deal with cytokines in vitro**. A-B.) Morphology shows that TGF-β induced a rapid proliferation of cells, and cells presented a spindle-like shape. The IFN-γ group presented a reduction tendency on cell adhesion, the shape of cells present suspended or polygonal, lose normal B16 cells morphousorm. When given TGF-β and IFN-γ at the same time, cells returned to normal B16 cell shape, and cells also grew. C.) The results by wound healing assay showed that TGF-β confronting IFN-γ can promote migration. To treat cells only by IFN-γ inhibited cells migration. D.) Based on the Transwell invasion assay, IFN can inhibit cell migration, and inhibit cell invasion through Matrigel, and TGF-β has the opposite effect on cells to IFN-γ, and may have also an activity for inhibiting the IFN-γ activity, so that the cells migrate and invade.

We investigated whether TGF-β can promote tumor cell proliferation or induced apoptosis, and whether IFN-γ can inhibit this tumor cell proliferation. In addition, we examined whether TGF-β can fight the inhibition effect of IFN-γ in the tumor cell when TGF-β and IFN-γ were administered at the same time in (the T and I group). A similar growth curve resulted for both the T and I group and the control group despite (no cytokines) were applied to the latter, providing growth opportunities for the cells under IFN-γ treatment. A morphology test also shows that when TGF-β induced a rapid proliferation of cells, the cells presented a spindle-like shape. On the other hand, the IFN-γ group presented a reduction tendency on cell adhesion, with the shape of the cells being suspended or polygonal. When administered with TGF-β and IFN-γ at the same time, the cells returned to their normal B16 cell shape (Figure 3A and 3B).

The results of the wound healing assay also showed that TGF-β confronting IFN-γ can promote cell migration. Treating cells with IFN-γ alone inhibited cell migration. Further experiments showed that IFN-γ can inhibit cell migration and invasion. This result was obtained through Matrigel as analyzed by Transwell invasion assay. TGF-β has the opposite effect on cells and may also possess the characteristics that inhibit IFN-γ activity. These lead to cell migration and invasion (Figure 3C and 3D).

### The lever of IFN-γ/TGF-β plays a new role in the activity of melanoma invasion

**Figure 4:**
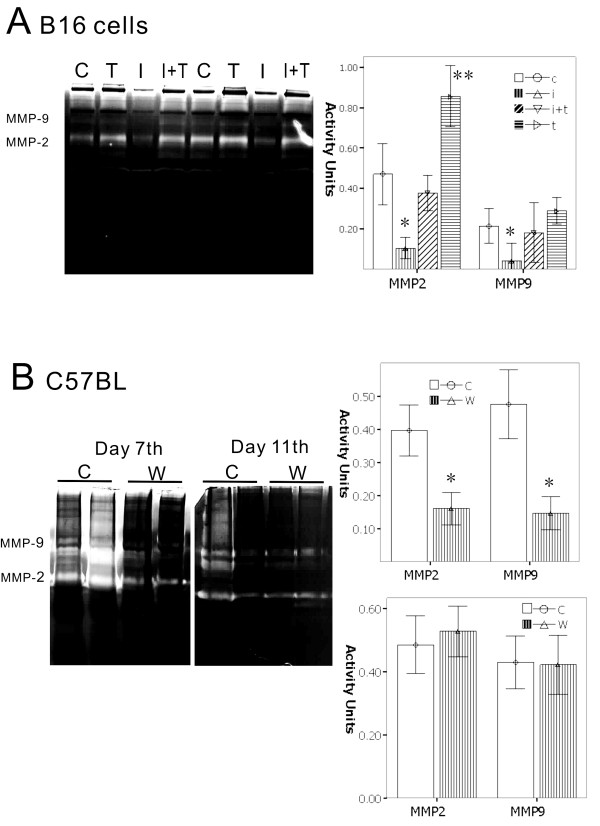
**To verify whether TGF-β and IFN-γ can enhance melanoma cells invasion by gelatin zymography assay analyzed *in vitro *and *in vivo***. A.) B16 cells treated by cytokines, show that IFN-γ can reduce the activity of MMP-2 and MMP-9, which are key modulators of tumor invasion. On the other hand, TGF-β can enhance the activity of both MMP-2 and MMP-9, giving TGF-β and IFN-γ. At the same time, TGF-β confronted IFN-γ to recover the activity of MMPs, and performed increasing activities on MMP-2 and MMP-9. B.) *In vivo *animal experiments also showed that there are significant features in day 7; the wound group had significantly lower activities of MMP-2 and MMP-9 compared with the control group from, 30% to 50%, respectively. By day 11, there was no significant difference in the activity of MMP-2 and MMP-9 between the wound groups and the control group. (*, *p *< 0.01)

To verify whether TGF-β and IFN-γ can enhance melanoma cell invasion, gelatin zymography assay was used. In the *in vitro *analysis, IFN-γ can reduce the activity of MMP-2 and MMP-9, which are the key modulators of tumor invasion. On the other hand, TGF-β can enhance the activity of both MMP-2 and MMP-9. At the same time, TGF-β confronted IFN-γ to recover the activity of MMPs, and increased the activity of MMP-2 and MMP-9 in the T and I group. *In vivo *animal experiments also showed that there are significant features on day 7, as the wound group had a significantly lower MMP-2 and MMP-9 activity as compared to the control group, from 30% to 50%, respectively. By day 11, there was no significant difference in the activity of MMP-2 and MMP-9 between the wound group and the control group (Figure 4).

**Figure 5:**
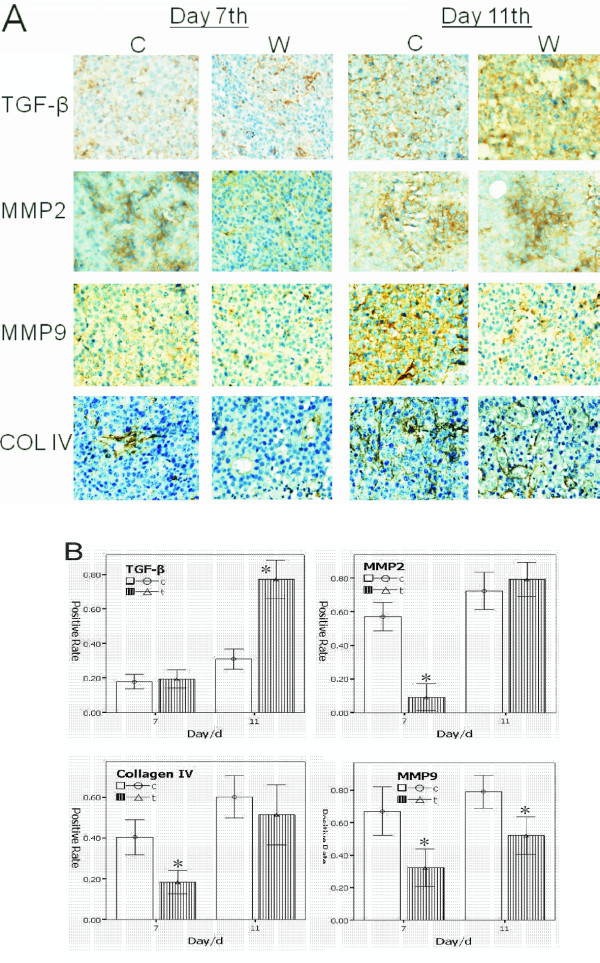
**Based on wound model described above, the tumor sample's immunohistochemistry analysis showed that, the TGF-β positive cells both in the wound group and the control group day 7 presented weak expression**. On day 11, the wound group presented significantly strong expression of positive cells higher than the control group. The positive cells of MMP-2 and MMP-9 show the same tendency as the results in the zymography, but when the TGF-β up-regulated expression, the activity of the state of MMP-2 and MMP-9 were restored from inhibiting to the highest expression. COL IV is an important extracellular matrix, and the percentage of positive cells in the wound group found on day 7 had a lower expression compared with the control group. However, in day 11, reflected in the control group with similar results, which show that both MMPs and extracellular matrix plasticity and inflammation will continue to dampen demand in the early phase, and reach to the latter phase. This is because the cytokines such as TGF-β, play new roles on tumor cells to escape the shackles of inflammatory factors, access to the growth, and progression. A.) The positive cells are stained in brown. B.) The positive percent of cells, *p *< 0.01 marked by *.

Immunohistochemistry analysis showed that the TGF-β positive cells in the wound and the control groups at day 7 presented weak expression; on day 11, the wound group presented significantly strong expression of positive cells higher than the control group. The positive cells of MMP-2 and MMP-9 show the same tendency from the results in the zymography. However, when the TGF-β up-regulated the expression, the activity of the state of MMP-2 and MMP-9 is restored to inhibiting the highest expression, which are similar to *in vitro *results. Collagen IV (COL IV) is an important extracellular matrix, as tumor cells were used to build the early vascular structures, and play important roles in tumor growth, angiogenesis, as well as cell invasion and metastasis [9,10]. We analyzed COL IV on days 7 and 11. The percentage of positive cells in the wound group found in day 7 also had a lower expression compared with the control group. However, in day 11, the positive cells had similar results with the control group. This shows that with both MMPs and extracellular matrix plasticity, inflammation will continue to dampen demand in the early phase, and reach the latter phase, as cytokines such as TGF-β play new roles on tumor cells to escape the shackles of inflammatory factors, access to growth, and progression (Figure 5).

### Investigation of the antagonism between IFN-γ and TGF-β by IFN-γ injection model in vivo

**Figure 6:**
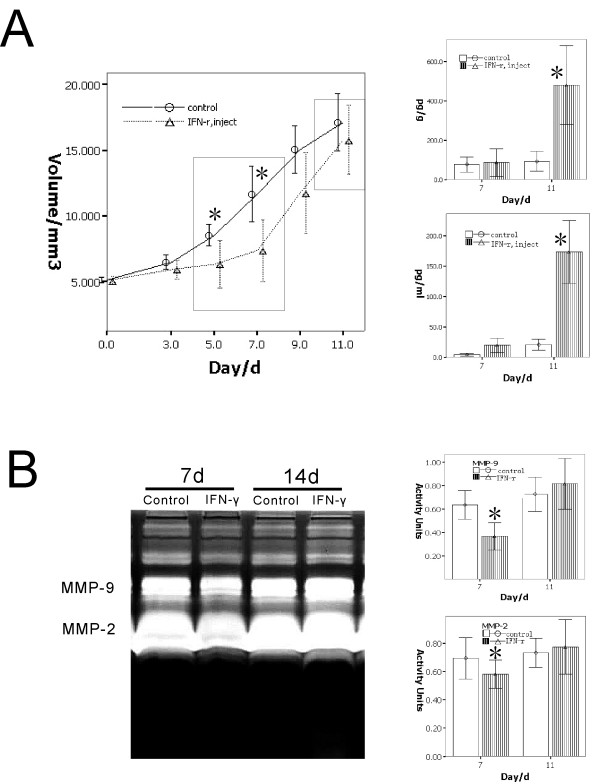
**Determination of the effect of IFN-γ injection on the tumor via tail-vein to validate the IFN-γ released from the wound model**. A.) The tumor growth curves showing the double-phase in the IFN-γ injection group, the inhibition phase, and the inhibition missing phase. In the inhibition missing phase, the level of TGF-β increased significantly in the IFN-γ injection group as compared to that in the control (marked by *, *p *< 0.05). B.) The activity of MMP-2 and MMP-9 as detected by the gelatin zymography analysis showing the decrease in the inhibition phase of the IFN-γ injection group and the significant increase in the inhibition missing phase as compared to the control group (marked by *, *p *< 0.05).

To investigate the process in which IFN-γ plays an important role in the process of wound inhibition on tumor, a validation experiment was done. We injected IFN-γ into the tail-vein (injection group) to mimic the inflammatory factors from the wound. The results show a similar effect on both the wound group and the injection group. The tumor growth curve showed two phases similar to the curve of the wound group: the inhibition phase (days 5 to 9) and the inhibition missing phase (after day 9). In the inhibition phase, there are no differences on the level of TGF-β between the injection group and the control group. However, in the inhibition missing phase, the level of TGF-β increased significantly both in the serum and the tumor of the injection group as compared to the control group (Figure 6A).

The activity of MMP-2 and MMP-9 in the tumor tissue was also detected by the gelatin zymography assay. In the inhibition phase, IFN-γ slowed down the activity of MMP-2 and MMP-9, which was not observed in the control group. On the other hand, in the inhibition missing phase, the TGF-β expression was high, which enhanced the activity of both MMP-2 and MMP-9 to confront the IFN-γ in the IFN-γ injection group. These findings were not observed in the control group (Figure 6B).

## Discussion

To understand the role of inflammation in cancer evolution, it is important to understand the nature of inflammation and how it contributes to physiological and pathological processes such as wound healing and infection. While this phenomenon has been discussed for more than 100 years, recent data have redefined the concept of inflammation as a critical component of tumor progression. Many types of cancer arise from inflammation [1-3,11-13]. While we are particularly concerned with inflammation promoting the formation of tumors, it should be noted that inflammation, especially in the wound healing process, has many similarities as well as differences with tumor formation. First, the inflammation in the process of wound healing involves the formation of granulation tissues, and the stromal cells of the components need to be built. Likewise, it involves the process of angiogenesis. Both the formation of granulation tissues and angiogenesis are similar to the formation of tumor stroma [14], as both of them have similar existence in the cytokines network [15]. Second, wound healing is controlled and limited. However, we found that the tumor was uncontrollable, especially in cell proliferation and angiogenesis [1,2,16-18]. In the initial stages of inflammation, the body's normal regulatory mechanisms control the wound-healing process and tissue growth. This normal regulatory mechanism does not exist in a tumor.

When the tumor and wound are in one body, the inflammation of the wound interacts with the tumor. The interaction depends on the distance between them. If the tumor is far from the wound, the interaction is mainly effected by the inflammatory factors of the serum. Inflammation in the process of wound healing under the body's normal regulation, which may be in the form of cytokines or inflammatory factors in the serum delivered to the tumor, is observed. On the other hand, tumor cells can also transmit molecular signals to the region of the healing wound to affect the process of inflammation and wound healing. For instance, although the immune system in tumor patients after surgery is usually abnormal, the surgery wound would still heal well. Furthermore, the residual tumor tissue promotes wound repair and the healing process. To investigate the interaction between the tumor and the inflammatory process in wound healing, we established a stab wound on tumor-bearing mice, and expanded it everyday to ensure that wound healing remains in the early stage.

Melanoma is a leading cause of cancer-related deaths worldwide through the aggressive and complex ways of angiogenesis [19-22]. Melanoma cells have a strong cytokine-secreting ability and complex signal regulatory networks [23,24]. The B16 melanoma cells came from the C57BL mouse, which has a normal immune system [25]. We used this animal model to determine the interaction between wound healing and cancer.

The first observation of our study is on the early stages of the wound. The tumor growth slowed down significantly until the wound was within the seven-day period of the model. We named this the tumor inhibition phase. At this phase, inflammatory factors played important roles in interfering with tumor cell proliferation by blood circulation. One of these factors is IFN-γ. Our data suggest that the serum and tumor had high levels of IFN-γ. IFN-γ is secreted from activated cells such as Th1 CD4+ T-helper cells into the tumor microenvironment. This enhanced antitumor immune responses and in turn induced the activation of macrophage cytotoxic activity [7,26,27]. IFN-γ increased susceptibility to apoptosis through Fas activators and cytotoxic chemotherapies in many cell types, including melanoma and colorectal carcinoma [28-30]. Through interactions with p53 and the inhibitor of apoptosis, XIAP, the ISG product XAF1 may allow APO2L/TRAIL to fully activate downstream caspases [31,32]. IFN-γ can up-regulate tumor-associated antigens, carcinoembryonic antigen, and TAG72 both in vitro and in vivo [33]. IFNs can also inhibit angiogenesis by altering the stimuli from tumor cells and by directly inhibiting endothelial cells. Endothelial cells are inhibited in motility; they undergo coagulation necrosis in vitro, while the inhibition of angiogenesis occurs in vivo within 24 hours of tumor cell inoculation. Suppression of bFGF, also known as FGF2, is correlated with reduced vascularization and tumor growth [34]. The following are the reasons that accounted for our results. First is the tendency of the wound to release IFN-γ into the blood, transport it into the tumor, inhibit tumor growth, and promote tumor necrosis. The wound group was significantly affected as shown by the reduced tumor volume. The cross-section revealed a high percentage of necrosis.

Interestingly, the persistence of the wound after seven days (the earlier phase) showed a weakened influence on the tumor. The tumor volume began to increase gradually as compared to that in the control group. This was followed by the tumor size approaching or exceeding the size of that in the control group. In other words, in the first seven days after the wound secretes IFN-γ and the other factors, the tumor cells were inhibited. After seven days, no reduction in the level of IFN-γ was observed. This was confirmed when TGF-β was tested in serum or tumor. The trend was higher. As such, IFN-γ did not inhibit the tumor cells. We named this the "inhibition missing" phase.

Perhaps a series of cytokines could explain the contradiction of the inhibition missing phase. The cytokine TGF-β was detected in the tumor tissue in the wound group after day 7, and should have been released into blood circulation which would likely restore the growth of the tumor cells. To test whether TGF-β had contrary effects on IFN-γ, an in vitro assay was done. This proved that TGF-β has antagonism with IFN-γ, can resume the growth of tumor cells, migration, and invasion; it can also lead to the situation wherein IFN-γ reduces the activity of the tumor cells' MMPs. In this situation, the tumor cells restored growth and invasion, and avoided the inhibition of IFN-γ. The validation experiment in vivo also presented a similar effect on the tumor by IFN-γ injection. The level of TGF-β also increased significantly in the inhibition missing phase. Furthermore, the activities of MMP-2 and MMP-9 were also enhanced in the inhibition missing phase as compared to those in the inhibition phase.

TGF-β is an important mediator of tumor progression, which likewise regulates cell proliferation, migration, and invasion; it is an important cytokine involved in a variety of biological processes [35,36]. We detected VEGF-a, bFGF, and other cytokines both in the serum and tumor tissue. However, only the expression of TGF-β up-regulated in the "inhibition missing phase," and was positively correlated to an increase in tumor size. The in vitro data proved that TGF-β can confront IFN-γ so that the tumor cells can restore proliferation and migration, and that it has the ability to resume invasion and the activity of the MMPs. The validation data in vivo also showed similar effect and phenotype. The IHC data also support this conclusion, as well as point out that Col IV is likewise regulated by the TGF-β/IFN-γ level.

In conclusion, the study has proven that when the wound and the tumor exist at the same time, there will be a new balance between TGF-β and IFN-γ. The wound, through the secretion of IFN-γ, interferes with the growth of the tumor cells and inhibits the tumor for a short period. Some tumor cells, through unknown mechanisms, use TGF-β against the IFN-γ effect in the restoration of tumor proliferation, invasion, and migration. As for the source of TGF-β, we speculated that the tumor cells mainly came from inflammatory factors such as IFN-γ adaptability to up-regulated expression, or were derived from the interaction between the tumor cells and the stromal cells. This needs further research to be conclusive. However, this study has proven that at least, in the interaction between tumor and inflammation by wounds, the existence of a new balance between TGF-β and IFN-γ not only contributes to the understanding of how tumor cells adapt to the inflammatory factor, but also provides a new basis to analyze the effects of the inflammatory process on tumors. This study also provides a reference to tumor surgery, especially in post-operative residual tumor assessment.

## Competing interests

The authors declare that they have no competing interests.

## Authors' contributions

MY carried out the animal experiment, participated in the design of the study. ST carried out all *in vitro *cell experiment, participated in the design of the study and draft the manuscript. LYX participated the animal experiment and carried out morphological observation. ZN and GQ carried out the immunohistochemical staining. ZD performed the statistical analysis. QS and NC participated in the study design and coordination. LY carried out the data collection. SB carried out the design of the study. All authors read and approved the final manuscript.
